# Conditions Affecting the Performance of Peripheral Vein Cannulation during Hospital Placement: A Case Study

**DOI:** 10.1155/2017/9748492

**Published:** 2017-11-07

**Authors:** Monika Ravik, Anton Havnes, Ida Torunn Bjørk

**Affiliations:** ^1^Department of Nursing Science, University of Oslo, PB 1018, Blindern, 0315 Oslo, Norway; ^2^Faculty of Health and Social Studies, University College of Southeast Norway, Porsgrunn, Postboks 235, 3603 Kongsberg, Norway; ^3^Centre for the Study of Professions, Oslo and Akershus University College of Applied Sciences, Postboks 4, St. Olavs Plass, 0130 Oslo, Norway

## Abstract

Learning practical nursing skills is an important part of the baccalaureate in nursing. However, many newly qualified nurses lack practical skill proficiency required to ensure safe patient care. The invasive skill peripheral vein cannulation (PVC) is particularly challenging to learn and perform. This study explored conditions influencing nursing students' learning and performance of the technical implementation of a PVC during their clinical placement period. A qualitative and descriptive case study design with two students in Norway practicing PVC during their clinical placement was conducted. One student who mastered the vein cannulation was compared with one student who did not. Data were collected in late 2012 using multiple data sources: semistructured interviews, ad hoc conversations, and video recordings. Video recordings of the two students' cannula implementations were used to help clarify and validate the descriptions and to identify gaps between what students said and what they did. Thematic analysis of the transcribed text data enabled identifying themes that influenced skill performance. There were two overall themes: individual and contextual conditions influencing the technical implementation of a peripheral vein cannula. These findings were evaluated in terms of Benner's work on scientific and practical knowledge, defined as “knowing that” and “knowing how.”

## 1. Introduction

Registered nurses must be qualified in practical skill performance to ensure quality in patient care [1]. Practical nursing skills are complex tasks involving technical aspects, theoretical and practical knowledge, caring intentions adjusted to both patient and environment, and ethical and moral considerations [2]. Although practical skill learning is a core component in nursing education [3, 4], many newly graduated nurses lack proficiency in practical skills [2, 5, 6]. Peripheral vein cannulation (PVC) is one of the most frequently performed invasive skills by nurses working in hospitals, with up to 70–80 percent of hospitalised patients requiring medication delivered through a vein cannula [7, 8]. PVC is considered the skill most difficult to master [5], especially the technical part of the cannula insertion: how to insert the cannula into the vein [9]. Mastering the technical part of PVC is important for completing other elements of the skill and undertaking it in a satisfactory manner [9].

There is a concern about the risk of patient harm associated with a lack of skill proficiency in registered nurses [e.g. 6, 10, 11]. Vein inflammation, phlebitis, is an example of a consequence caused by poor vein cannulation [10–13]. Phlebitis contributes to clinical symptoms such as pain and local infection signs [11]. A phlebitis caused by catheter related infections has a risk of progressing into a bloodstream infection [12], increasing inpatient days in hospital [14]. Inadequate skill performance may also have consequences for the nurses, frustration, making mistakes, and time pressure [6], and is a reason why newly qualified nurses leave the profession [15].

For many years, simulation has been used in nursing education to help students improve their practical skills learning and performance and to prevent errors [1, 16]. In many nursing programs the use of simulation has increased to support students' skill learning [17]. However, the varied and complex situations that appear with patients and equipment are limited in simulation training, impeding practical skill learning [18]. Simulation-based learning provides students with initial skill proficiency, and being able to perform and develop their practical skill performance in the clinical setting is a desired outcome of simulation [1].

Clinical practice is a major component of the nursing education curriculum and is considered a very important learning environment for the development of practical skills competence [19–22]. Experiencing practical skills performance on patients is beneficial to understanding practical skills more thoroughly than the understanding what is only obtained from simulation-based learning [23]. This concurs with a sociocultural learning perspective that assumes that learning cannot be understood without reference to the social and cultural context in which it is embedded [24]. Registered nurses' provision of high-quality feedback is important as it is associated with student satisfaction and better performance [25, 26]. Learning supported by hints from more knowledgeable others is termed scaffolding [27]. Learners are able to master more in collaboration with more experienced others than when they are alone [24]. Repeated performance in similar situations is also identified as a particularly important element in students' learning [28]. Benner [28] argued that each experience of skill performance builds on the previous one and that scientific knowledge will be refined and expanded as the learner gains experience. Learning in the clinical setting is, however, challenging due to several constraints, such as missed learning opportunities [9, 29] and inadequate support and respect [22].

Learning how to insert a vein cannula is the most challenging skill taught in nursing school [30]. Several investigators have focused on methods used for teaching PVC [31–34]. Knowledge is still lacking on how students may best acquire technical mastery in PVC during a clinical placement period. Development of knowledge related to all aspects that influence students' learning of PVC is needed, as well as establishing the mostly valuable learning experiences for students. Nursing education must be concerned with conditions influencing practical skill learning [7]. In this study the aim was to explore conditions that affected students' learning of the technical part of PVC in the clinical setting.

### 1.1. Aims and Research Questions

We report here one part of a larger study on nursing students' learning of PVC in nursing education. In this study we aimed to explore two student cases concerning PVC learning and performance during a clinical placement period. By comparing one student who mastered the technical implementation of the vein cannula with one student who did not, we aimed to describe in detail the conditions that influenced their ability to master the technical implementation of a peripheral venous cannula on patients in the clinical setting. This exploration may in turn afford an understanding of challenges that students encounter in their learning processes in the clinical setting and can form a basis for improving students' practical skill learning during their clinical placement. In previously published articles from the larger study, we have reported on transfer of skill learning (PVC) from simulation to the clinical setting [9] and learning actions that students were engaged in during simulation-based PVC learning [35]. Because of challenges found in the previous studies related to learning and performances of the technical implementation of the vein cannula, we have in the current research explored conditions influencing students learning and performances of this particularly part of the skill during a clinical placement period. We formulated two research questions:

1. How do different conditions influence the two students' success in mastering the technical aspects of cannula implementation?
2. How do the two students experience their own mastery of technical implementation in PVC?

## 2. Methods

### 2.1. Design

The present research is based on extensive fieldwork. An exploratory and descriptive case study design was used, which aims to describe and compare issues within and across cases, thereby generating a rich understanding [36]. The richness of case studies is related to the amount of particular and context (detail and contextualisation) that is possible when only a small number of cases are included and analysed [36]. Case study research is regarded as appropriate when gaining an in-depth understanding of individual participants' behaviour and experiences in a natural setting [37]. Two contrasting student cases with one student in each case were selected to provide in-depth knowledge about conditions influencing practical skill learning and performance during a clinical placement period. The research methods used were semistructured interviews, ad hoc conversations, and video recordings.

### 2.2. Sample and Setting

This in-depth case study of two nursing students is part of a larger study conducted for a doctoral dissertation. The larger study included nine participants enrolled in the third semester of a baccalaureate in nursing. The students had their first clinical placement period in a hospital, and they were by the main researcher video recorded in a total of 38 attempts (3–6 attempts by each student) of PVC during their practice period. After completed clinical placement, the analysis of video recordings of all nine students' performances of PVC revealed that two students differed greatly in the technical implementation of the vein cannula. Student A succeeded in four out of five video-recorded attempts, and Student B failed in all six video-recorded attempts. The other seven students were more similar in their performances, with a few successes and more failures. Students A and B were inductively selected as two individual cases to answer the research questions, meaning that they were chosen without theoretical or conceptual implications, but based on the students' contrasting mastery of the vein cannulation [38].

No student had previous experience with PVC on patients. Each student had one main supervisor, a registered nurse who had the major responsibility for mentoring and evaluating during the clinical placement period, but also several other registered nurses mentored the students when they performed the skill because they had the primary responsibility for following up the patient who needed a PVC. All the registered nurses were experienced in PVC, but none of them were educated in supervision. As of today, there is no formal regulation of registered nurses pedagogical competence when they supervise students during clinical placement in Norway [39]. The main researcher encouraged the registered nurses to supervise in the same way as when they were not observed. The nurses stayed close to the student and patient when the students penetrated the skin and vein and inserted the vein cannula. Both students were able to take care of the patient when they cannulated vein and asked the patient about pain and discomfort. The registered nurses were also alert to the comfort of the patients, asking and observing the patient for pain and discomfort. Each student also had a teacher contact from the academic setting who was responsible for midpoint and final evaluation of the student. The teacher was not involved in the study, and video recordings were planned to not coincide with meetings set up with the teacher.

The study was conducted in two acute medical wards in one hospital in the southern part of Norway. The students were informed about and recruited to the project in the academic setting before they started their clinical practice period. The registered nurses and patients involved in the study were individually informed about the project prior to the data collection. Registered nurses were informed by the main researcher, and the patients were informed by the students or registered nurses responsible for the care of the patient who needed a PVC. Patients who needed a PVC, and agreed to let students perform the skill on them, were asked if they were interested in joining the study. Video recordings were not performed during physician visits or other informational meetings.

### 2.3. Data Collection

Data were collected in late 2012. The research field was entered for nine weeks to enable in-depth investigation of nursing students' learning and performance of PVC during a clinical placement period. Multiple data sources, individual semistructured interviews, ad hoc conversation, and video recordings, were used because they promote a comprehensive and deep understanding of an investigated phenomenon from different perspectives [36, 37]. Semistructured interviews and ad hoc conversations were conducted to generate knowledge and insight into students' experiences and perspectives in vein cannulation [40]. Video recordings were used to explore details of the technical implementation of vein cannulation that are otherwise difficult to obtain [41] but also to make a comparison between text and video data to reveal discrepancies between self-reported data and actual performance. It is common to find some gaps between what is said and done [42].

Video recordings of Student A included 5 attempts of PVC (76 minutes) with 5 different patients, and video recordings of Student B included 6 attempts of PVC (71 minutes) with 4 different patients (twice, Student B had two attempts on the same patient). Both students were video recorded during their first PVC attempt. All the attempts of PVC were performed on the veins of patients' hand and arm (basilica, cephalic, metacarpalis, and dorsalis). Ad hoc conversations with the students took place before and after almost all the video recordings. No ad hoc conversations were conducted after the first PVC attempt (the students were formally interviewed at this time) or between the first and second attempts of PVC on the same patient. The students were interviewed twice. The first interview was performed at the beginning of the clinical placement, after the first PVC attempt. The second interview was conducted at the end of the clinical placement after the last video-recorded PVC attempt and based on video-stimulated recall. Prior to answering questions, students watched the video recording of one of their last PVC attempts. This provided students with the opportunity to remember aspects of and talk about details in their skill performance and learning [43].

Interview guides were developed for both interviews covering the following themes: positive and challenging experiences related to the cannula implementation on patients and similarities and differences between skill performance in the skills centre and clinical setting. In the first interview, an additional theme covered the students' preparation for skill performance. Ad hoc conversations aimed to make the informants feel comfortable, create a relationship of trust between the researcher and the informants, and allow the students to discuss aspects of PVC in an informal way. Interviews and ad hoc conversations were tape-recorded. Four interviews and sixteen ad hoc conversations were transcribed verbatim.

### 2.4. Ethical Consideration

This research was performed according to the ethical principles in the Declaration of Helsinki [44] and approved by the Norwegian Social Science Data Service (NSD), University College, and hospital. All the participants were informed both orally and in written way about the project. Informed consent was obtained from the two students, registered nurses, and patients who were video recorded. To enhance patients' anonymity, their faces were not video recorded. In a multibedded patient room, the video camera's zoom function was used to protect other patients not included in the study.

All participants were informed that they were free to withdraw from the study at any time, and the patients were clearly informed that they could stop both the student's skill performance and the video recording. The patients were adults and elderly. One of the patients had a hearing impairment, one had recently received pain medication, and one patient was treated by CPAP (continuous positive airway pressure), but they were awake, oriented, and responsive. Students did not perform PVC in patients that were dying, uneasy, and unconsciousness, who refused to let students practice PVC on them, or in emergency situations. None of the patients were obese or visibly dehydrated. Patients were not first assessed by mentoring nurses to ensure that they had visible and palpable veins. The video recordings show that Student B was not exposed to more challenging veins to cannulate than Student A.

Although the students were mentored by nurses during their skill performances, the main researcher continuously assessed the students' performances for the necessity to interrupt the students' skill performances (because of pain and discomfort). Patients must be protected from potentially harmful consequences that can affect them as a result of their participation in the study [45]. This is a balancing act when exploring actions in a naturalistic setting: when to let things pass when ethical aspects are not violated and when to stop the action and thereby influence the phenomenon under exploration. Even if the vein was missed Student B was by patients and nurses provided positive feedback about the attempt of PVC, for example, being both calm and careful during the skill performance. After the first unsuccessful attempt of vein cannulation the patients encouraged Student B to try one more time.

To maintain confidentiality, students' age, sex, or medical ward types were not given. To reduce the introduction of bacteria into the patient environment the video camera was disinfected before entering the patient room.

### 2.5. Data Analysis

In this study all the data were explored with a focus on the technical implementation of the vein cannula. Interview and ad hoc conversation transcripts were initially analysed within each case, followed by a cross-case analysis [36, 37]. An inductive thematic analysis inspired by Malterud's [46] principles of systematic text condensation guided the analysis of the transcribed texts, which was conducted in the following steps:

1. All transcripts were read thoroughly to form a general view of the texts, and central themes were defined.
2. Meaning units related to each theme were identified and coded.
3. The codes were sorted into code groups, and the meaning in each code group was condensed and summarised.
4. Descriptions were developed reflecting the influential themes concerning learning and performance of the technical part of a PVC.

After analysing the transcribed texts, the technical implementation of the PVC was systematically explored in the 11 video recordings. The seven steps defined as the technical implementation of PVC [9] were based on steps in guidelines for PVC developed by Cappelen Damm [47] (Table 1).

We described events during the seven technical steps on the video recordings related to the themes identified in the text data. A cross-case analysis provided description of the similarities and differences found between the two cases, generating a holistic view of students' learning of vein cannula implementation [36].

## 3. Results

The study findings are organised into two overarching themes: (1) individual and (2) contextual conditions influencing the technical implementation of PVC. The former theme comprises three subthemes: search for scientific knowledge, embodied knowledge, and gaining self-confidence. The latter theme comprises two subthemes: consistency across the learning arenas and feedback from supervising nurses.

### 3.1. Individual Conditions Influencing the Technical Implementation of PVC

The conditions described in this section concern the students as learners. There are major differences in the students' attitudes toward the role of scientific knowledge and embodied knowledge. This section also shows how the success or failure of cannula implementation affects the development of the students' self-confidence.

#### 3.1.1. Search for Scientific Knowledge

The interview data showed that students had different views on the need for knowledge related to the insertion of a PVC. Student A stated in the first interview the importance of achieving a certain level of scientific knowledge about PVC in order to perform the skill properly: “I have read all the theory concerning the guidelines, which has covered most things, such as complications, like, for example, phlebitis and thrombophlebitis, and I have got a good understanding of what it is.” Repeated readings were noted as important to acquire relevant knowledge. Difficult words and expressions were written in a separate book that could be easily kept in a uniform pocket. The student discussed both the actual skill performance and how to safeguard the patient, and knowledge was highlighted as an important factor in providing good care.

In both the interviews, Student B reported reading very little theory about the procedure during clinical placement. Studying scientific knowledge was not a priority. In the first interview, the student stated, “I have to admit that I have only read the guidelines. I have not had the energy to read additional theory related to the guidelines or any other literature.” Primarily, the student's attention was concentrated on the skill performance and less toward learning knowledge to safeguard the patient.

#### 3.1.2. Embodied Knowledge

The students' understanding of handling the cannula during skin and vein penetration differed, which led to differences when inserting the cannula into the patient's vein. In the first interview, Student A knew the cannula should be positioned at different angles during the vein insertion, “When penetrating the skin and vein, I place the cannula at an angle of 25–30 degrees. Immediately after the penetration, I decrease this angle to a few degrees. I use these angles so as not to perforate the vein.” The angle of the cannula became integrated in the student's way of handling the cannula, whereby the student no longer had to reflect or think about the angle of the cannula. Already in the first interview, the student said, “I get this feeling that I have gone as deep as I should. I can feel it in my fingers; like a blind script.” The student sensed that the angle of the cannula depended on the depth of the patients' vein, which varied for each patient.

In the first interview, Student B also expressed that the cannula should have different angles during vein implementation but said several times during clinical placement that it was difficult to sense these angles. In the second interview, the student said, “I do not think I have come very far in the technical implementation of the cannula. I have perhaps gained a bit more security in how to penetrate the skin and vein, but it is more like guessing about the angle needed to insert the cannula into the vein after penetration.” The student's guessing was directly connected to a lack of sensing in the fingers.

#### 3.1.3. Gaining Self-Confidence

The interviews showed that the students' success or failure in cannula implementation and self-confidence in handling the technical aspect of the skill were interrelated, and this was illustrated by the differences between the two students. Prior to the first interview, Student A had three positive experiences with cannula implementation (one in the clinical setting after two successful implementations during simulation). Being successful with the implementation at this early stage provided a positive feeling. Regarding the inspirational impact of success, the student said, “Had I missed three times, I would have really been nervous to try the next time.”

Conversely, Student B was unsuccessful in implementing the cannula in all the video-recorded PVC attempts. The student incrementally lost belief in his/her own ability, moving from a belief and hope of being successful to a personal feeling of failure. Several times during the clinical placement, the student explained the importance of succeeding in the cannula implementation to give a good impression of his/her own ability. In the fourth ad hoc conversation, the student said, “It is very important what other people think about me and what I am doing. If I feel that I do not give a good impression, I become self-critical real fast, which constrains my learning process.” The student's attention was directed toward his/her own achievement as well as being assessed and judged by others.

Being successful in implementing the vein cannula in the simulation and in the clinical setting may build self-confidence, as expressed by Student A in the second interview: “Even if I have missed a couple of times, I believe it is due to other circumstances. It has nothing to do with how good I am or not.” The student's initial self-confidence was retained even when unsuccessful cannulations occurred. Interestingly, while Student B failed the cannula implementation in the first attempt on a patient, the student said it provided a positive experience. The student believed in being close to success and expected to succeed the next time. Each time the student failed, self-confidence decreased. In the second ad hoc conversation, after four failed attempts of cannulation, the student expressed a personal defeat, “I admit that not being able to master the skill starts being a burden.” The student's lack of self-confidence resulted in a growing distancing in skill training and thereby learning opportunities. In the second interview, the student said, “I do not want to try to insert the cannula into a patient's vein if there is no realistic chance to actually hit the vein and implement the cannula.”

### 3.2. Contextual Conditions Influencing the Technical Implementation of PVC

This section describes the two major contextual conditions that impacted the students' learning of the technical part of the PVC: similarity versus dissimilarity in the use of guidelines across learning arenas and differences in feedback from nurses in the two medical wards.

#### 3.2.1. Consistency across the Learning Arenas

The video recordings showed that Student A performed the practical skill in the same way in the clinical setting as previously learnt in the skills centre. Similarities between the learning arenas were considered valuable. In the first interview, Student A said, “The procedure is very similar because they are following the same guidelines, and it feels safe because it is great when our training or education is similar to what we have to do in the clinical setting.” When differences occurred between the two learning arenas, the student wished to continue executing the skill in the same way as learnt during simulation: “I'd rather use a syringe with 10 ml sodium chloride to flush the cannula than a smaller one, because that was what we learnt in the simulation training.” The student's preferences were acknowledged and favourably looked upon by the nurses, and the student could continue to use the equipment as preferred.

Student B encountered a ward where all nurses used a different technique during vein cannulation than the one learnt in the skills centre. In the second interview, the student commented, “The nurse taught me to flush the cannula with sodium chloride at the same time as I advanced the cannula into the vein. I tried to do it that way, but always messed it up because the cannula became unstable when I changed my grip.” The student found the new technique implementing the cannula more challenging than the technique previously learnt during simulation.

#### 3.2.2. Feedback from Supervising Nurses

In both the first interview and fourth ad hoc conversation, Student A said that the nurse had to make the student aware of crucial elements regarding the technical implementation of the vein cannula. The student found it difficult to remember all steps needed to insert the cannula into the vein, especially when there were long periods between each attempt. In the fourth ad hoc conversation, the student said, “I had forgotten that I should be aware of blood in the flashback chamber. If the nurse had not reminded me, I would probably have inserted the cannula too deep.” In the first two video recordings, we saw that nurses guided Student A to insert the cannula into the vein before the stylet was withdrawn. During the other video-recorded attempts, the student performed this crucial step independently.

Conversely, the video recordings of Student B documented the lack of feedback given to the student or hints on how and when to advance the cannula before removing the stylet, although this was an obvious and repeated challenge for this student. In the third ad hoc conversation, after several attempts, Student B was still sure that the cannula was inserted into the vein at every attempt, “It is emphasised in the guidelines that the cannula has to be advanced a few millimetres before withdrawing the stylet a bit after the flashback of blood in the flashback chamber. I am 99% sure that I follow the guidelines, but I think I insert the cannula too much and then perforate the vein.” The video recordings showed that the student never performed this particular crucial step and failed the cannula implementation each time. The vein was missed as the stylet was removed before the cannula was secured into the vein. The nurses were only seen as observers in this step of the cannulation.

## 4. Discussion

In this qualitative study, learning and performance of peripheral vein cannulation was influenced by individual and contextual conditions, and acquisition and use of knowledge were the main issues. To advance the understanding of how knowledge impacted vein cannulation in patients, the meaning of these findings and their implications will be discussed in light of Benner's [28] work on scientific and practical knowledge: what she defined as “knowing that” and “knowing how.” In vein cannulation, “knowing that” can be seen as scientific knowledge, such as verifiable knowledge on which guidelines for practical skills are grounded, but also theory underpinning varied challenges and issues in different practical situations. “Knowing how” is practical knowledge, which is shown in doing practical tasks, such as managing different techniques [28]. Implementing a PVC includes both “knowing that” and “knowing how.”

The students had contrasting attitudes toward acquisition of scientific knowledge. Student A found a wider spectrum of knowledge about PVC important, such as danger of complications, as this knowledge gave a broader perspective on the performance itself. Student B did not prioritize the acquisition of any knowledge about PVC during clinical placement. In principle, students do not need to know all knowledge related to a practical skill to perform the skill successfully and correctly, but some scientific knowledge is crucial to performing the skill correctly. Knowledge about hygienic principles is, for example, necessary to avoid inappropriate consequences, such as infections [48]. Being familiar with scientific knowledge, such as the guidelines, does not automatically make the student skilled in the skill performance [28], as seen in the performance of Student B.

Both students described a sensing in the fingers as important for the vein cannulation. Student A was not experienced in cannula implementation, yet quite early during the clinical placement sensed that different angles of the cannula were necessary to penetrate the skin and vein. In contrast, Student B had difficulty sensing the varied angles of the cannula, and the cannula was placed into the vein by guesswork. We suggest that both “knowing that” and “knowing how” is important for success in vein cannulation. The importance of scientific knowledge is also supported by Dall'Alba and Sandberg [49], who suggested that integrating new knowledge with previously learnt knowledge contributes to a deeper understanding of practical knowledge. This is embodied knowledge, which is knowledge whereby the body knows how to behave [28]. Knowledge that is embodied constitutes an essential dimension of professional skill performance and its development [49].

In Benner's [28] work on novice-to-expert development, repeated experience is emphasised as necessary to become skilled and develop embodied knowledge. In this study we found that experience alone was insufficient to become skilled in vein cannulation. Student B had several PVC attempts but never learnt to use the right angles needed to insert the vein cannula. We propose that a lack of both scientific and embodied knowledge about the varied angles needed in vein cannulation prevented the student from developing appropriate “know how” on vein cannulation. Student A, conversely, had scientific knowledge that became embodied into skillful practice and there was no need for guesswork.

How students succeeded in vein cannulation influenced their self-confidence in skill performance. Self-confidence refers to the strength of belief and judgment about one's capabilities to achieve a goal [50]. Student A succeeded in vein cannulation and became self-confident. A self-confident student who trusts his or her capabilities in performing successful vein cannulations can focus on new learning opportunities with PVC, instead of worrying about reasons for unsuccessful cannulations. Conversely, Student B had limited growth in vein cannulation as the vein was repeatedly missed. A lack of mastering the vein cannulation negatively influenced the student's self-confidence. The student withdrew from opportunities to practice PVC due to fears of what others' thought about unsuccessful cannulations. A barrier in the student's learning was thereby created. Killam and Heerschap [51] also found that students were scared of what nurses thought about their behaviours. The students did not admit that they lacked the “know how” expected from them and avoided asking questions. They performed even if they lacked self-confidence.

Being exposed to similar guidelines in the two learning arenas facilitated the learning process, while inconsistency in steps and the sequence across the skills centre (simulation) and clinical settings impeded the learning process. This is consistent with recent study findings in which similar guidelines in the two learning arenas enabled students to transfer practical skill from simulation to the clinical setting [9]. However, guidelines must be adapted in local practice to secure performances that meet the individual patient's needs [52]. It is therefore unrealistic to suggest that the clinical setting should harmonize practice or guidelines with the guidelines that students are introduced to in the academic setting. Educational institutions may, however, consider using a broader approach in learning the critical steps in many practical skills. Marton [53] has suggested that students should be prepared for differences appearing in varied situations to understand a task from a wider perspective. Students encountering different ways of performing a task may extract meaning and choose alternatives appropriate in varied situations. A closer cooperation between the academic and clinical setting may uncover differences in the two learning arenas [54, 55] that can be incorporated into simulation-based learning to simplify the transfer process of practical skills to the clinical setting [53].

The students knew that the cannula had to be entered into the vein to be successfully implemented but found it challenging to apply this knowledge into their “knowing how.” The importance and difficulties applying scientific knowledge during practice were described by Katajavuori et al. [56] in a study on pharmacy students. Almost half of the students experienced challenges in applying pharmacological knowledge to patient counselling, resulting in unprofessional counselling. In nursing education, nurses are important supervisors and role models during nursing students' clinical practice [25, 26]. Helping students link scientific knowledge and action is a crucial part of nurses' scaffolding strategies; a more proficient person providing relevant feedback helps the student develop both intellectually and practically [24, 27, 57].

In this study, Student A was reminded that the cannula should be ensured into the vein before withdrawing the stylet; Student B was never helped to link such knowledge to action. Inadequate pedagogical knowledge required in supervision may be the reason for this lack of support [58]. Student B repeatedly engaged in an incorrect pattern of performance. The gap between the student's performance and the professional standard of PVC was not addressed by the supervisor [59]. This illustrates that a lack of implementation of “knowing that” into “knowing how” constrained the student's familiarisation with the technical part of PVC and the making of efficient decisions. The student persisted at a novice competence level [28]. We suggest that a lack of growth in vein cannulation can be quite destructive, leading to feelings of incompetence and a lack of professional identity development. This is in line with Carr et al. [60] who emphasised that successful learning and performance of peripheral vein cannulation were important to reduce failure and improve clinician and patient satisfaction, as well as increasing the life expectancy of the device used.

### 4.1. Limitations and Strengths

Only two cases of practical skill learning were investigated. However, the cases were contrasted and triangulation in data collection provided detailed knowledge about the conditions influencing the students' technical implementation of a peripheral vein cannula [36, 37]. The researcher with a video camera could be considered distracting or constrain the students' skill performances [41]. However, the reasons for one of the students missing the vein in each cannulation attempt cannot be connected to the video camera as the student thought the vein cannula was inserted into the vein every time.

## 5. Conclusion

This qualitative case study was based on two nursing students' different levels in vein cannulation mastery. Conditions influencing the two nursing students' different levels of mastery were described in detail. These conditions were both individual and contextual. Student A acquired a deep understanding of how to technically implement a vein cannula due to many parallel and positive influencing factors: the student was knowledgeable, was helped by nurses to implement scientific knowledge into action, was offered the opportunity for repeated practice with similar guidelines, and developed self-confidence in the cannula implementation as practical skill competence increased. In contrast, Student B's opportunity to obtain the relevant “know how” in cannula implementation was disturbed by conditions such as insufficient scientific knowledge, having to learn the technical implementation of the cannula in a new way, nurses who did not provide feedback on how to place the cannula into the vein, repeated missing of the vein, and a lack of building embodied knowledge and self-confidence.

Further research is needed to explore students' learning and performance of technical aspects of other practical nursing skills. We must uncover whether the technical aspect of skills in general is challenging in nursing students learning. Furthermore, students' learning conditions in simulation-based learning and the interaction between nurses and students during students' clinical placement must be further explored to ensure that students are supported to meet the learning objectives needed to be successful in practical nursing skills.

### 5.1. Relevance to Clinical Practice

This study has shown the importance of both theoretical and practical knowledge to learn and advance in vein cannulation and that students should acquire theoretical knowledge needed to gain skill proficiency. Registered nurses responsible for mentoring students during their clinical placement period should make sure that students acquire knowledge necessary to learn and advance in their skill performances. The registered nurses should also help students to apply in the clinical setting what they previously have learnt with simulation training. Having to learn the vein cannulation in a new and different way in the clinical setting is disadvantageous because it contributes to relearning and prolonged learning processes [61]. The supervisors, the more proficient others, should look at themselves as change agents, who are capable of making a difference for the learners, helping them to develop learning and understanding [62]. Hattie [62] termed this as “Know thy impact,” meaning that supervisors need to refocus their efforts to ensure that any action taken has maximum impact on the individual's learning and achievement. This study suggests that registered nurses responsible for supervising students should be pedagogically educated to proficiently undertake their supervision role; they should be knowledgeable in how they can use themselves as “change agents,” so they can help students to advance in their skill learning and performances during their clinical placement period.

## Figures and Tables

**Table 1:** Illustration of the procedural steps of PVC. The seven steps concerning the technical implementation of vein cannulation are highlighted.

Peripheral venous cannulation: procedural steps [47], operationalised by Ravik et al. [9]
(1) Prepare all equipment
(2) Clean hands
(3) Ensure patient identity
(4) Select a vein
(5) If required, clip hair around the insertion site
(6) Apply a tourniquet to the chosen limb
(7) Assess the patient's vein while selecting appropriate site
(8) Release the tourniquet
(9) Ensure that the skin to be anaesthetized is clean and dry
(10) Select device
(11) Clean hands
(12) Clean and establish workspace
(13) Empty equipment on cleaned workspace
(14) Wet sterile swabs with a 0,5% chlorhexidine solution
(15) Prepare a needleless bung with 0,9% sodium chloride
(16) Prepare an extension tubing
(17) Prepare a needleless bung with heparin 100 IE/ml
(18) Make the patient comfortable sitting or lying down
(19) Place a dressing towel under the patient's arm
(20) Clean hands
(21) Clean the patient's skin and vein with a 0,5% chlorhexidine solution
(22) Clean the skin and vein once more with a 0,5% chlorhexidine solution
(23) Allow the skin to air-dry for minimum 30 seconds
(24) Put on gloves
(25) Reapply the tourniquet
(26) *Apply manual traction a few centimetres below the proposed insertion site*
(27) Select site of insertion
(28) *Use a three-point cannula grip*
(29) *Insert the cannula through the skin*
(30) *Once flashback is seen in the flashback chamber, advance the cannula a few millimetres into the vein before withdrawing the stylet a few millimetres*
(31) *Slowly advance the cannula forward into the vein in a smooth movement*
(32) Release the tourniquet
(33) *Apply firm pressure to the vein above the distal cannula tip*
(34) *Remove the stylet*
(35) Discard the stylet into sharps container
(36) Connect an extension tubing
(37) Attach a needleless bung with 0,9% sodium chloride
(38) Flush the cannula with 0,9% sodium chloride
(39) Observe for signs of swelling or leakage
(40) Ask patient about discomfort or pain
(41) Flush the cannula with heparin 100 IE/ml
(42) Cover the insertion site with a transparent dressing
(43) Write date/initials on dressing
(44) Apply bandage
(45) Discard the waste
(46) Remove gloves
(47) Clean hands
